# Discontinuation of dental care and systemic diseases of persons in need of long-term home care – an observational study from the InSEMaP project with German health insurance claims data

**DOI:** 10.1186/s12877-026-07702-5

**Published:** 2026-06-02

**Authors:** Espen Henken, Hans-Helmut König, Alexander Konnopka, Anja Behrens-Potratz, Stefanie Schellhammer, Petra Schmage, Thomas Zimmermann, Claudia Konnopka, The InSEMaP-Group, Thomas Beikler, Başak Erakın, Roschan Farhumand, Maren Heyke, Alena Koenig, Sarah Porzelt, Mohamad Ramadan, Seyit Sahan, Martin Scherer, Regina Semmelhaack, Peter Stratmeyer, Lydia von Palubitzki, Doniyor Yuldashev

**Affiliations:** 1 Department of Health Economics and Health Services Research, University Medical Center Hamburg-Eppendorf, Martinistr. 52, Hamburg, 20246 Germany; 2 Department of Psychiatry and Psychotherapy, University of Leipzig Medical Center, Leipzig, Germany; 3 Faculty of Business and Social Sciences, Department of Nursing and Management, Cooperative Process Management in Social and Healthcare RTC (KoPM-Zentrum), Hamburg University of Applied Sciences, Hamburg, Germany; 4 Department of Health Care Research and Innovation, Deutsche Angestellten-Krankenkasse-Gesundheit (DAK-G), Hamburg, Germany; 5 Department of Periodontics, Preventive and Restorative Dentistry, University Medical Center Hamburg-Eppendorf, Center for Dental and Oral Medicine, Hamburg, Germany; 6 Department of General Practice and Primary Care, University Medical Center Hamburg-Eppendorf, Hamburg, Germany

**Keywords:** Dental service use, Systemic diseases, Home care, Long-term care, Claims data

## Abstract

**Introduction:**

Impaired oral health poses a risk factor for several systemic diseases. The aim of this study was to investigate associations between the discontinuation of regular dental care use and several systemic diseases among persons with an incident long-term home care need.

**Methods:**

Using German health and long-term care insurance data from the DAK-Gesundheit of persons aged ≥ 60 years, we partitioned the observational period into a baseline (2015–2016), an exposure (2017–2018), and a follow-up period (2019–2020). We selected persons who regularly visited the dentist in 2015–2016 with incident and lasting need for long-term home care from 2017 on. We compared persons who discontinued dental care (DDC group) with those who continued to regularly visit the dentist (CDC group) regarding the occurrence of several systemic diseases. We applied entropy balancing and analyzed the probability for each systemic disease using weighted logistic regressions.

**Results:**

We selected *n =* 11,767 persons in the CDC and *n =* 1,477 in the DDC group. We found slightly higher odds for dementia in the DDC than the CDC group, but there were no significant differences between groups regarding the occurrence of: type 2 diabetes, cardiovascular disease, chronic obstructive pulmonary disease, pneumonia, oral cancer, and rheumatoid arthritis.

**Conclusion:**

The discontinuation of regular dental services was associated with dementia. However, the studied conditions and the impact of a discontinuation of dental care on general health likely progress slowly and the direction and cause of the association remains unclear.

**Supplementary Information:**

The online version contains supplementary material available at 10.1186/s12877-026-07702-5.

## Background

Multiple studies have found associations of impaired oral health and systemic diseases [1]. Poor oral health, in particular periodontal disease or periodontitis, might be a potential risk factor for diseases like rheumatoid arthritis (RA [2]), chronic obstructive pulmonary disease (COPD) and pneumonia [3], and cardiovascular disease (CVD) or associated events [4]. For some conditions, like type 2 diabetes and periodontal disease, findings even suggest a bidirectional association [5]. Pathways for these associations are still inconclusive [1, 6], but proposed mechanisms include inter alia genetic and environmental factors, bacteremia and microbial dysbiosis, and comprised immune responses [7] and the oral biofilm might play a crucial role [8]. For example, periodontal disease might either affect Alzheimer’s disease by intensifying brain inflammation or because associated bacteria might enter the bloodstream and thus the brain [9].

As regular dentist visits are important to maintain good oral health [10], multiple studies from different countries investigated the association of different types of dental services and health conditions. Regarding CVD, observational studies on different populations found associations between different types of dental services or lack thereof and atherosclerosis [11], CVD and CVD events [12–18], or related outcomes [19]. Similarly, observational studies have associated different types of dental care use with a lower diabetes risk [12, 13], albeit with ambiguous results [12], a lower hospitalization risk for type 2 diabetes patients [19], a lower incidence of pneumonia among hemodialysis patients [20], and fewer hospitalizations due to pneumonia [14]. Additionally, a meta-analysis suggested a potential association of periodontal treatment and fewer COPD exacerbations [21]. Moreover, a meta-analysis of observational studies found an association of a lack of dental visits and incident oral cancers [22]. First evidence from RA patients did not indicate an association of periodontal treatment and hospitalizations or medical costs [19]. Regarding dementia or cognitive decline, several observational studies indicated an association with dental service use [13, 23, 24], although inconclusive evidence exists [24–26].

In a German study, chronic diseases were more common for those with a long-term care need than for those without [27]. Moreover, persons in long-term care, particularly those receiving home care, utilize dental services less often than those not in long-term care [28]. Persons receiving home care showed worse oral health than those in nursing homes [29] and an incident home care need was associated with an increased risk of discontinuing the use of dental services [30]. Hence, persons with an incident home care need who stop going to the dentist might be particularly at risk for impaired oral health and thus potentially for several diseases. Therefore, we hypothesized that discontinuing regular dentist visits with the onset of a home care need might affect the probability of these diseases.

## Methods

We conducted a retrospective cohort study with health and long-term care insurance claims data from 2015–2020 provided by the DAK-G, one of Germany’s largest statutory health insurance companies. The study describes the second research question of working package 2 of the InSEMaP (Interaction of Systemic Morbidity and Oral Health in Ambulatory Patients in Need of Home Care) project. A description of deviations from the research protocol [31] can be found in the supplementary materials.

Persons selected by the DAK-G were continuously insured with the DAK-G, ≥ 60 years old in 2017, without long-term care need in 2015–2016, and had a first recorded long-term care level in 2017. As described in the German Social Code Book (SGB), §15 SGB XI [32], a long-term care need is assessed by a standardized examination which evaluates the ability of performing activities of daily living and the functional status. There are five care levels – higher levels describe higher degrees of impairments of abilities and functional status. Long-term care services can either be provided at a patient’s home (home care) or in a nursing home (inpatient care).

We split the observational period into a baseline (2015–2016), an exposure (2017–2018), and a follow-up period (2019–2020). In the baseline, all included persons had at least one routine dental visit (*routine visit)* in each year. A routine visit was defined as a dentist visit with a claim of service codes 01 or 04 of the German uniform fee for dental services (BEMA [33]). BEMA 01 is reimbursed for a routine check-up while BEMA 04 describes a periodontitis screening. Thus, all included persons were presumably regular dental care users prior to becoming care dependent. Medically necessary dental services and routine examinations are covered by the statutory health insurance in Germany [34]. While long-term care recipients might organize a visit to the dentist themselves, barriers regarding transportation or suitable facilities [35] might render this difficult. Whereas cooperation contracts of dentists and nursing homes have been indicated to increase dentist visits to nursing homes [36], home visits to individual residencies are difficult and time consuming [35] and persons in need of home care might lose contact to a dentist. We assigned persons with at least one routine visit in 2017 and 2018, respectively, to the continued dental care group (CDC) and those without any routine visit in 2017–2018 to the discontinued dental care group (DDC). Both groups were compared regarding the occurrence of several systemic diseases in the follow-up period. A description of the study design can be found in supplementary Fig. 1.

Selecting from *n =* 45,677 persons with a care level in 2017, we applied several exclusion criteria: *n =* 11,350 persons died during the study period and were excluded as a complete observation was necessary to assess several relevant variables. Moreover, we excluded *n =* 19 persons with missing data for population density (used for risk adjustment). Additionally, *n =* 11,668 persons without a routine visit in each baseline year were excluded. Moreover, *n =* 392 persons lost their care level during the exposure or follow-up period and were excluded. Because we focused on persons with a home care need, we also excluded *n =* 5,414 persons who transitioned to nursing homes (indicated by any claim for inpatient long-term care services and including those with prolonged short-term care stays). While short-term care was not assessed as inpatient care service as it is usually used to overcome care shortages in an home care setting, all persons with short-term care for more than 56 days per year were excluded because it is only reimbursed for 56 days per year. Thus, the selected sample of persons with home care need included those with a continuously recorded care level from 2017 to 2020 and without recorded inpatient care services (except short-term care). Lastly, we excluded *n =* 3,590 persons in the control group who did not have a routine visit in either 2017 or 2018. See supplementary Fig. 2 for an illustration of the selection process.

### Variables

We used the group assignment CDC and DDC as independent variable in all analyses. We assessed the following systemic diseases during baseline and follow-up, for which impaired oral health, particularly in terms of periodontal disease or periodontitis, might be a risk factor (as well as bidirectional links): oral cancer [37], CVD [4], dementia/cognitive impairment [38, 39], type 2 diabetes [5], COPD and pneumonia [3, 40], and rheumatoid arthritis [2]. We acknowledge that this is not an exhaustive selection of diseases and that existing evidence might be inconclusive.

We identified the presence of each condition using data from the outpatient medical care (only those recorded as *gesichert*: secure and Z*ustand nach*: condition after*)*, inpatient hospital, outpatient hospital (only secure and condition after), and medication healthcare sector records. We used the ICD-10-GM and ATC codes as shown in supplementary Table 1 and assessed whether they were present per baseline and follow-up quarter in each health sector, respectively. Second, we assessed whether a condition was present per person and year. To increase validity of the diagnoses recorded in the claims data, a condition was present if an outpatient diagnosis with the respective ICD-codes was recorded in at least two quarters or an in- or outpatient hospital diagnosis was recorded in at least one quarter per year. Quarters were not required to be consecutive and diagnoses could originate from different or the same provider. We applied this base scenario for oral cancer, CVD, and pneumonia. For COPD, dementia, rheumatoid arthritis, and diabetes we applied additional criteria, which are described in the supplementary materials. Lastly, we defined a systemic condition to be present in the respective period if it was present for at least one year within baseline or follow-up period, respectively. Conditions in the exposure period were not assessed as these variables were used neither for risk adjustment nor as outcomes. The occurrence of each systemic disease in the follow-up period served as dependent variables. We also assessed the number of diseases – the sum of these binary variables.

We prepared several other variables for risk adjustment: The population density (on January 1st 2017) of each person’s place of residency, the number of dental and routine dental services during baseline, the first recorded care level (levels for 4 and 5 were collapsed in one category due to their rare occurrence), and the number of days passed in 2017 until onset of the care level. Furthermore, we summed each person’s healthcare costs in each sector during baseline – outpatient hospital, dental services, medical appliances, medical devices, inpatient hospital, home health care services, medication, outpatient medical care, and rehabilitation. To mitigate the impact of extreme outliers on the risk adjustment, all costs were winsorized at the 99th percentile. Lastly, we assessed 22 medication-based comorbidities during baseline [41] if a medication with a relevant ATC-code for the respective comorbidity was recorded.

### Risk adjustment

To reduce the risk of baseline differences between groups to confound the group comparison, we applied entropy balancing [42]. Entropy balancing is a weighting method that weights the individuals in the control group (CDC) in such a way that the mean, variance, and skewness for all utilized covariates closely match those in the treatment group (DDC). Compared to other common weighting or matching procedures like inverse probability weighting or propensity score matching, it produces a superior balance between samples [43]. We used the following variables for entropy balancing: Gender, age (in 2017), population density, number of dentist visits and routine visits, occurrence of each systemic disease during baseline, care level at onset and number of days until onset, healthcare costs per sector during baseline, and medication-based comorbidities [41]. Medication based comorbidities that overlapped with the focal systemic diseases assessed in this study (cancer, CVD, dementia, diabetes mellitus, respiratory illness – asthma, COPD –, and rheumatologic conditions), as well as those that occurred in less than 1% of the cases in either group (CDC or DDC) were disregarded. We used the entropy balancing weights in all analyses.

### Statistical methods

We used weighted logistic regressions for the analysis of the occurrence of each disease (binary) and a weighted Poisson regression for the analysis of the number of diseases. As all different outcomes were a surrogate for the same hypothesis, we adjusted for multiple testing with the Holm-Bonferroni procedure [44], adjusting from α = 0.05.

We calculated five sensitivity analyses: First, the care level at onset and the days until the care onset could not be assessed before the start of the exposure (as the care onset was during 2017 not necessarily at the start of 2017) and might theoretically be affected by the exposure. Hence, all analyses were recalculated without balancing for care variables. Second, we applied a stricter criterion for the discontinuation of dental care by only considering persons in the DDC group without any dental visit (instead of focusing on routine visits). Likewise, any dental visit per year was sufficient for persons in the CDC group in this analysis. Third, all persons who did not have a routine visit in 2017 were still included in the CDC group because in the year with an onset of home care, those persons might have to re-arrange regular dental service due to changed circumstances. Fourth, we shortened the follow-up to one year to rule out effects of the Covid-19 pandemic and adjusted the selection criteria accordingly. Fifth, we calculated an unadjusted, unweighted analysis to assess the impact of entropy balancing. We conducted all analyses using R (version 4.40) and entropy balancing with Stata 16 (StataCorp, College Station, TX). Additional methodological information can be found in the supplementary materials.

## Results

Overall, we selected *n =* 1,477 persons in the DDC and *n =* 11,767 persons in the CDC group. A description of both groups before and after applying entropy balancing can be found in Table 1.

**Table 1:** Descriptive statistics before and after EB

Baseline: 2 years	DDC group (*n =* 1,477)	CDC group (*n =* 11,767)	
		Before EB	After EB
Female [%]	74.68	72.82	74.66
Age [mean]	80.85 (7.67)	79.28 (7.16)	80.83 (7.67)
Population density: medium [%]	45.02	45.08	45.03
Population density: dense [%]	38.32	37.9	38.34
Dentist visits [count]	4.7 (2.63)	5.23 (2.87)	4.71 (2.64)
Routine dentist visits [count]	2.48 (0.74)	2.86 (0.92)	2.48 (0.74)
Dementia [%]	8.94	9.06	8.94
Cardiovascular disease [%]	27.35	28.82	27.37
Rheumatoid arthritis [%]	3.99	4.89	4
Pneumonia [%]	4.33	4.62	4.34
Diabetes [%]	32.43	33.86	32.45
Chronic obstructive pulmonary disease [%]	7.45	8.19	7.45
Oral Cancer [%]	0.34	0.56	0.34
Care level at onset of care: level 2 [%]	53.69	51.28	53.66
Care level at onset of care: level 3 [%]	13.2	9.93	13.2
Care level at onset of care: level 4 and 5 [%]	2.84	0.76	2.84
Number of days until long-term care onset in 2017	175 (109)	172 (108)	175 (109)
Costs during baseline (€)			
Outpatient hospital	25.52 (132)	39.44 (160)	25.62 (132)
Dental services	577 (680)	599 (684)	577 (680)
Medical appliances	482 (1,108)	735 (1,289)	484 (1,109)
Medical devices	1,135 (2,334)	1,343 (2,452)	1,135 (2,335)
Inpatient hospital	5,581 (9,872)	6,089 (9,832)	5,583 (9,875)
Home health care services	710 (3,006)	585 (2,739)	710 (3,006)
Medication	2,602 (4,399)	3,221 (5,478)	2,606 (4,413)
Outpatient	2,130 (1,523)	2,643 (1,741)	2,133 (1,526)
Rehabilitation	402 (1,241)	476 (1,268)	402 (1,241)
Medication-based comorbidities (≥ 1% per group) [%]			
Acid related disorders	53.15	58.38	53.17
Bone diseases (osteoporosis)	9.95	11.52	9.96
Epilepsy	3.66	4.56	3.66
Glaucoma	12.53	12.94	12.54
Gout, Hyperuricemia	15.64	15.76	15.65
Hyperlipidemia	39.81	42.74	39.83
Intestinal inflammatory diseases	1.56	1.95	1.56
Iron deficiency anemia	4.94	5.64	4.95
Pain	55.11	60.76	55.14
Parkinson`s disease	6.64	9.95	6.66
Psychological disorders (sleep disorder, depression)	33.04	36.62	33.08
Psychoses	7.65	7.69	7.66
Thyroid disorders	26.95	29.16	26.97

Parentheses depict standard deviations. Comorbidities that overlapped with systemic diseases of interest were not used in entropy balancing (cancer, cardiovascular diseases, dementia, diabetes mellitus, respiratory illness [asthma, COPD], and rheumatologic conditions). HIV, Migraines, Tuberculosis occurred in less than 1% in at least one of the groups and were not used

*EB* Entropy Balancing, *DDC* Discontinued dental care, *CDC* Continued dental care

The sample was about 80 years old and predominantly female. Most of the selected persons had care level 2 at onset (51.3% CDC group, 53.7% DDC group) and the most common of the studied systemic diseases were CVD (28.8% CDC group, 27.4% DDC group) and diabetes (33.9% CDC group, 32.4% DDC group). Slight differences between groups were negligible after applying entropy balancing. However, regarding the care level, the progression during the observational period slightly differed between groups. While after weighting approx. 2.8% of both groups were in care level 4 and 5, this differentiated to 4.5% (CDC) and 6.1% (DDC) at the start of the follow-up and to 8.8% (CDC) and 12.7% (DDC) at the start of the third quarter of 2020 (see supplementary Fig. 3 for an illustration of the care level progression).

The results of the main analysis can be found in Table 2. After adjustment for multiple comparisons, the only significant group difference between DDC and CDC groups was regarding dementia – the probability for having dementia in the follow-up was higher in the DDC than the CDC group.

**Table 2:** Regression results

Disease	OR/IRR	Statistic	*p*	Adj. *p*^a^
Dementia	1.3 (1.09; 1.56)	3.98	<.001	<.001
CVD	1.09 (0.93; 1.28)	1.46	.143	.86
Rheumatoid Arthritis	0.81 (0.54; 1.2)	−1.38	.167	.86
Pneumonia	1.09 (0.84; 1.41)	0.80	.425	1
Type 2 diabetes	0.96 (0.83; 1.11)	−0.70	.485	1
COPD	1.05 (0.84; 1.32)	0.51	.609	1
Oral cancer	1.17 (0.57; 2.4)	0.44	.663	1
Number of diseases^*^	1.06 (0.99; 1.13)	2.26	.024	.167

*COPD* Chronic obstructive pulmonary disease, *CVD* Cardiovascular disease, *OR* Odds ratio, *IRR* Incident rate ratio

^*^Calculated using a weighted Poisson regression; all other estimates were calculated with weighted logistic regressions. Parentheses depict confidence intervals built with robust standard errors and with the respective α level that was used due to the Holm-Bonferroni procedure

^a^*p* was adjusted using the Holm-Bonferroni procedure

**Fig. 1:**
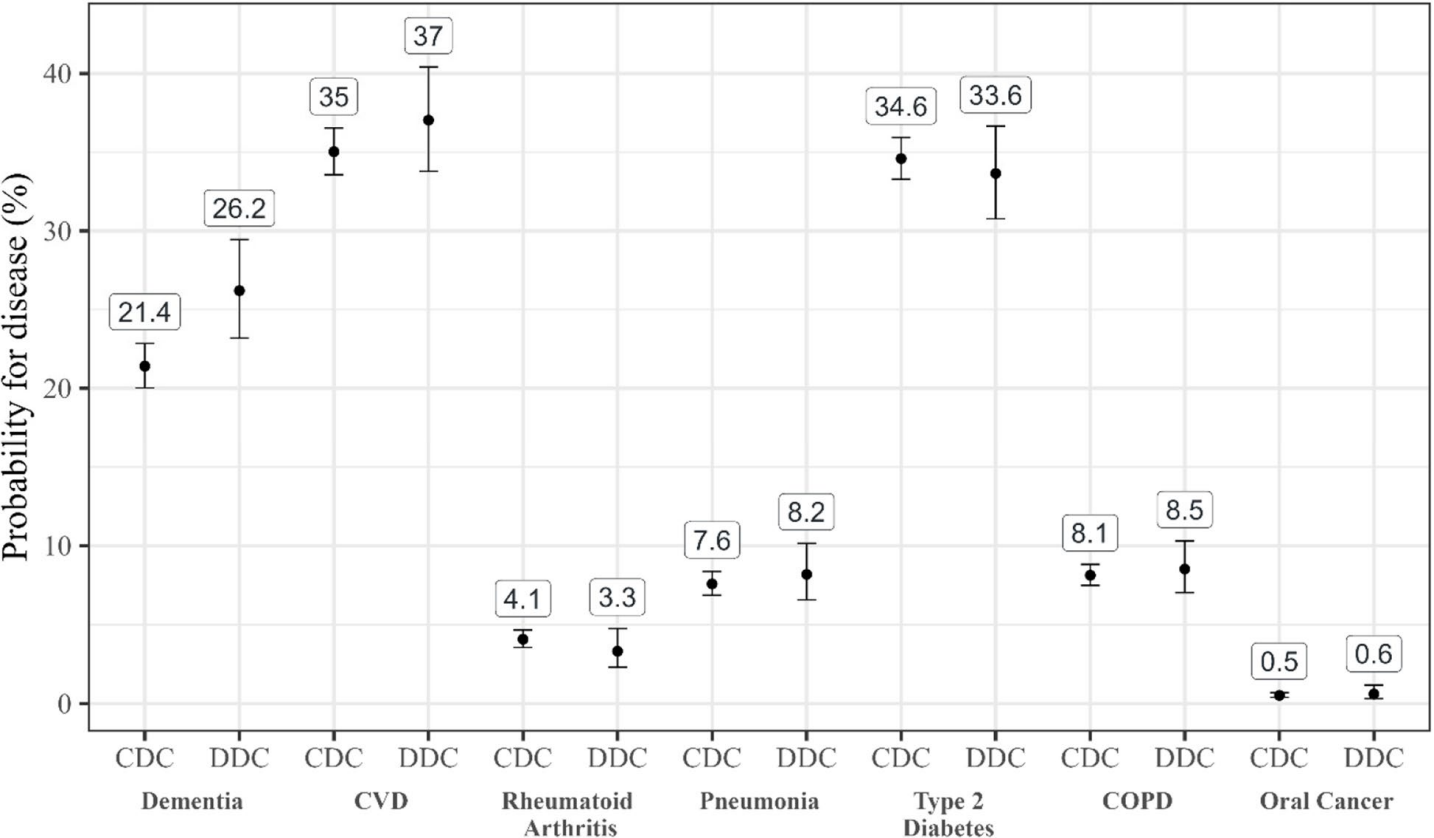
Predicted disease probabilities. Values depict predicted probabilities and confidence interval (using robust standard errors and Holm-Bonferroni adjusted alpha levels). COPD = Chronic obstructive pulmonary disease; CVD = Cardiovascular disease; DDC = Discontinued dental care; CDC = Continued dental care. All values were rounded to the first digit

Corresponding predicted probabilities are depicted in Fig. 1. They show a 21.4% (adj. confidence interval; CI: 20.0; 22.9) probability for dementia in the CDC and 26.2% (adj. CI: 23.2; 29.5) in the DDC group. The incident rate ratio for the number of diseases corresponds to a predicted count of 1.11 (adj. CI: 1.08; 1.14) in the CDC and 1.18 (adj. CI: 1.11; 1.25) in the DDC group.

**Fig. 2:**
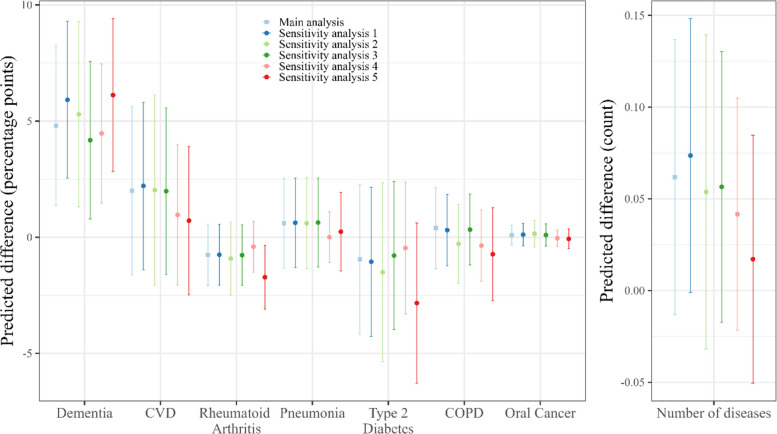
Sensitivity analyses. The figure depicts differences between discontinued and continued dental care groups in the predicted probabilities or counts and confidence intervals built with robust standard errors according to respective the alpha level adjusted with Holm-Bonferroni procedure. COPD = Chronic obstructive pulmonary disease; CVD = Cardiovascular diseases; Sensitivity analysis 1: Not balanced for care variables (sample size equal to main analysis); Sensitivity analysis 2: Stricter criterion for discontinuation (no dental services at all; sample size CDC group, *n =* 13,173; sample size DDC group, *n =* 1,045); Sensitivity analysis 3: Less strict criterion for continuation of dental services (sample size CDC group, *n =* 13,343; sample size DDC group, *n =* 1,477); Sensitivity analysis 4: one-year follow-up (sample size CDC group, *n =* 13,882; sample size DDC group, *n =* 1,942); Sensitivity analysis 5: No risk adjustment (sample sizes equal to main analysis)

The sensitivity analyses are illustrated in Fig. 2. They show risk differences – the differences in the predicted probabilities of DDC and CDC group – and analogous differences in predicted counts. All sensitivity analyses led to similar results.

## Discussion

We conducted a retrospective cohort study using health insurance claims data. Among persons with an incident long-term home care need, we compared the occurrence of several diseases between those who continued to regularly go to the dentist and those who discontinued going to the dentist. Dementia was more common among those who discontinued going to the dentist, but the direction of this association remains unclear. Besides that, we found no significant differences regarding cardiovascular diseases, chronic obstructive pulmonary disease, type 2 diabetes, oral cancer, pneumonia, or rheumatoid arthritis.

Many other studies found associations of some kind of dental service use and several of the health conditions studied here (e.g., [12–14]). We only found associations with dementia, which was about 22% more common in the group that discontinued to go to the dentist compared to the group that continued to go to the dentist. This finding is plausible given the potential associations of oral health and cognitive impairment [e.g., 39]. Moreover, these results are in line with prior studies that found associations of dental service use and dementia [13, 23, 24]. One explanation might be worse oral health and periodontal health in the DDC group (due to a lack of regular dental care), which in turn might have affected brain inflammation and thus the development of dementia, particularly Alzheimer’s disease [9]. Additionally, other studies have shown associations of tooth loss and cognitive decline [25, 45] and group differences in the number of remaining teeth might also be an explanatory factor. Common explanations for associations of tooth loss and dementia include decreased nutrition and lack of neuronal feedback (both due to masticatory dysfunction) in addition to inflammation ( [45] but see also [46] for a critical review of these explanations). However, measures like the DMF-T index are not part of health insurance claims data and thus we could not access differences in number of teeth. It would also be possible that those who developed dementia during exposure period discontinued going to the dentist with a higher probability than those who did not develop dementia rather than vice versa. As dental service utilization for persons with dementia has been found to decrease [47], this is an alternative plausible interpretation. Consequently, this result has to be interpreted with caution. Increased periodontal disease exposure in the DDC group would be a pathway for several of the other conditions studied here, like rheumatoid arthritis [2] or CVD [4]. As no significant differences were found for these conditions, it becomes less likely that this pathway explains the obtained differences regarding dementia. Overall, we could not assess oral health or whether oral health was deteriorating. Limo et al. [48] found that fewer past dental visits amplified the negative association of suboptimal oral health and multimorbidity. However, without information on oral health, we could not determine whether a deteriorating oral health after discontinuing dental service use did not materialize or whether poor oral health did not affect general health to an identifiable degree for most investigated conditions. Moreover, while a systematic review of longitudinal studies found a positive impact of dental visiting patterns on oral health in general, evidence regarding periodontal health was scarce [10]. Two years might have been too short for discontinued regular dental care to affect oral health enough to in turn affect general health. Moreover, information on oral health behaviors such as tooth brushing was missing. This might distort the association of discontinued dental care and systemic health if oral health behaviors among those who regularly visit a dentist differ from those who do not.

While we did not find associations of dental visiting patterns and several health conditions, contrasting several prior studies, our study differs in several ways from those in the literature. Overall, there are country-specific differences in the healthcare systems, in the coverage of dental services, and also regarding overall dental insurance coverage. Our analysis also differed from many of the prior investigations with regard to the definition of the exposure, outcomes, or investigated population. Many other studies focused on effects of specific treatments like periodontal treatment [18, 19], dental prophylaxis [17], or regular professional cleaning [15]. Furthermore, other studies investigated various operationalizations of regular dental service use and found an impact of regular dental service use on health conditions – especially on CVD [11, 16, 20]. Distinguishing this from other studies, we investigated a discontinuation of regular dental attendance by selecting on prior regular usage, testing a change in service use. Outcome definitions also were heterogenous between studies – while we investigated the occurrence of diseases, other studies, for example, investigated hospitalizations due to a disease [14, 19]. Differences with regard to the investigated populations might also explain divergent results because we investigated newly home care dependent persons. Other studies did not particularly focus on care dependent persons or focused on vulnerable populations like hemodialysis patients [18, 20]. Furthermore, as a high morbidity was already present during baseline, it might have been difficult to differentiate the impact of discontinuing dental care on additional diseases amidst an already high morbidity.

Considering that a tendency towards utilizing preventive dental services might reflect an overall tendency to utilize preventive health services and health services in general, this might also explain a lack of differences for most of the studied diseases. Therefore, it might have been more likely for those who utilized dental services to obtain a diagnosis for their diseases and undiagnosed diseases might have been more common for those who discontinued to go to the dentist. Using claims data, for which diseases are usually selected by requiring diagnoses in multiple quarters (at least in outpatient setting), might have amplified this.

Overall, there are also mixed and thus inconclusive results with regard to, for example, periodontal treatment and CVD [49], which would be in line with the current investigation. Likewise, there are also are mixed results regarding dental visiting patterns and cognitive decline as two studies did not find an association for older persons [25, 26].

### Strengths & limitations

One of the study’s strengths was that by selecting persons with incident long-term home care need, we studied a population that might be particularly prone to discontinuing using dental services [30] or using them less frequently [28] and vulnerable to several systemic diseases. Moreover, we selected persons with a regular dental service use prior to becoming care dependent. Hence, all subjects had a similar history of dental service use for at least two years. This approach allowed that differences in the occurrences of systemic diseases could be associated with the subsequent continuation or discontinuation of dental service use providing a nuanced description of the associations of a change in regular dental service use. Moreover, few studies investigated associations of dental attendance with COPD or RA occurrence yet. Nevertheless, studies suggested associations of oral health or oral disease and these health conditions [2, 3], which warrant further investigations. Lastly, another strength was the rich data set, which allowed for a comprehensive balancing of several variables with regard to demography, health care and dental services, and morbidity.

However, we did not balance for variables observed during the exposure period, because variables that changed during exposure might at the same time have affected the exposure or been affected *by* the exposure. To illustrate, while a change in the care level could affect both, the probability to go to the dentist as well as the probability of some diseases, the care level might have been affected by the exposure itself as a lack of dental attendance has been associated with increasing care dependency [50].

Moreover, defining routine dental services predominantly based on BEMA 01, we could not differentiate why a dentist was visited because a check-up according to BEMA 01 can also be conducted when the dentist visit was symptom rather than check-up driven. The two-year exposure period might have been too short not only for an impairment of oral health to manifest, but also to identify changes in potentially slowly developing health conditions. Also, as we separated exposure and follow-up period, it would be possible that persons reinstalled a regular dental service usage during follow-up, which might have reduced the potential effect of not going to the dentist.

Furthermore, potential unmeasured confounding for example by lifestyle factors like smoking or the socioeconomic status limits the strength of the current results. Additionally, we only had access to population density as regional information. Other studies, however, indicated that dental service utilization might vary by federal state [51], especially between former East and West Germany [28], or discussed that dentist density might explain regional variations [51]. Thus, our study might have come short in addressing potential confounding by regional variations. Moreover, there might be misclassification bias of recorded diagnoses or claims. While validating outpatient diagnoses by using multiple occurrences is important and common practice in claims data analyses [52], misclassification remains possible. Misclassification might, for example, originated from under-diagnoses of stigmatized diagnoses [53] or off-label-use of drugs. Lastly, using data of a single statutory health insurance does not permit generalization to the general population [54] and excluding all persons who deceased during the study period further affects the generalizability of the study.

## Conclusion

We conducted a retrospective cohort study with German health insurance claims data investigating the associations between the discontinuation of regular dental service use and several systemic diseases in a sample with an incident home care need. While we found a slightly higher probability for dementia in those who discontinued regularly using dental services compared to those that continued regular usage, there were no differences for other systemic diseases. The results demand a cautious interpretation as the question of directionality regarding the association of discontinued dental service use and dementia could not be resolved. To further disentangle the associations of dental service and systemic diseases, prospective studies with long follow-ups could investigate the development of these diseases – especially those with few prior investigations. Special attention should be paid in research as well as health and dental care to particularly vulnerable groups like long-term home care dependent persons. Overcoming intersectoral borders care givers, general practitioners, and dentists should work together to address the increased risk for dementia and of losing contact to the dentist.

## Supplementary Information

Supplementary Material 1.

## Data Availability

The datasets supporting the conclusions of this article are owned by the German statutory health insurance DAK-Gesundheit. In this case, anonymous data were used. For data availability, researchers must conclude a contract with the statutory health insurance. The licensee is permitted to use the data for the purpose of the research proposal within their company, exclusively. Licensees are not allowed to pass the data to a third party, or to create software or databases except for scientific publications.

## Abbreviations

BEMA: Einheitlicher Bewertungsmaßstab für zahnärztliche Leistungen (German uniform fee for dental services)
CI: Confidence interval
CVD: Cardiovascular disease
CDC: Continued dental care
COPD: Chronic obstructive pulmonary disease
DAK-G: Deutsche Angestellten Krankenkasse-Gesundheit
DDC: Discontinued dental care
InSEMaP: Interaction of Systemic Morbidity and Oral Health in Ambulatory Patients in Need of Home Care
RA: Rheumatoid arthritis
