# Supplementary material for: Discontinuation of dental care and systemic diseases of persons in need of long-term home care – an observational study from the InSEMaP project with German health insurance claims data
Source: BMC Geriatr. 2026 Jun 2;26:796. doi: 10.1186/s12877-026-07702-5 (PMC13231774; doi:10.1186/s12877-026-07702-5)
Supplement: Supplementary file 1 — Supplementary Material 1. [file 12877_2026_7702_MOESM1_ESM.pdf]

Discontinuation of dental care and systemic diseases of persons in long-term home care

– an observational study with health insurance claims data from Germany

Supplementary Materials

Deviations to the research protocol

For transparency we note some deviations to the research protocol of the InSEMaP project [1]. First, the projected sample size of persons with an onset of a lasting need for long-term home care (n = 6,000) was substantially lower than the realized sample size. The main reason for this was that we adjusted the definition of "lasting need for long-term home care". Originally (in the projections and first data deliveries), this only included persons who had a continuous care level starting in 2017 and continuously received long-term home care services until the end of 2020. However, this is a rather narrow definition as it only describes those who receive long-term home care services without any interruption due e.g., a long hospital stay. Moreover, as a byproduct, this excluded persons who died or moved to nursing homes during the study period. Therefore, an additional data delivery was conducted by the DAK-G providing all persons with an incident need for long-term home care in 2017 (as defined by the first record of a care level in the observational period) irrespective of the reimbursed care services. Of these, we selected all those with a continuous care level and without any record of inpatient (i.e., nursing home) long-term care services as those with a need for home care. This provided a larger sample than projected.

Second, the original study design planned an individual three-year follow-up per persons starting on the day of the first recorded care level instead of a two-year follow-up starting at the beginning of 2019. To separate exposure and follow-up period, however, we adjusted the design as described in the methods section. This approach also had the advantage to operate on calendar-years and quarters instead of individual observational periods, which better align with claims data's billing cycles.

Third, contrary to the descriptions in the study protocol, we did not assess the severity of already existing diseases as many diseases are not recorded in the health insurance claims data in a way that permits to capture different degrees of severity. In particular, there would be

no option to uniformly access the severity across health conditions to allow for a joint analysis. Thus, we refrained from a detailed analysis of the diseases' severity.

Lastly, the selection criteria were adjusted to the modified observational periods ensuring that persons were alive, had a regular dental service use during 2015 and 2016, and in need of long-term home care starting from 2017 until the end of the observational period. Although not described in the protocol, persons with implausible (this applies for sensitivity analysis four) or missing data (regarding population density) were also excluded.

Additional information relating to the methods

We modified the base scenario and applied the following additional criteria to access COPD, dementia, rheumatoid arthritis, and diabetes: For COPD, following Buhl et al. [2] we only used outpatient data from pulmonologists. For dementia, we also considered dementia-specific medication [3]. Thus, in addition to the base scenario, we additionally defined dementia as being present in years with a recorded anti-dementia medication in two different quarters as well as in years with an outpatient diagnosis in one quarter and a medication in another. We increased precision for RA by applying the base scenario and additionally only defining RA to be present in years with at least one disease-specific medication [4]. We applied the first 6 steps of the procedure of Reitzle et al. [5] to differentiate between type 1 and type 2 diabetes (we only used the latter). For this, we first selected all years using the base scenario and additionally, all years with only one quarter with an outpatient diagnosis if a relevant medication was reimbursed in the same year and then differentiate between diabetes types using the described procedure [5].

In all weighting procedures, we used the population density of the place of residency at the start of the exposure period. It had three levels: low, medium, and high density, which we dummy-coded for balancing. Overall, n = 19 persons (< 0.2% of the final sample) were ex-

cluded due to missing population density information. For more information on how the regional information of population density was merged to the health insurance claims data, see the supplementary material of Henken et al. [6].

In the fourth sensitivity analysis, we shortened the follow-up to only include 2019. Thus, in contrast to the main analysis, persons who died during 2020 were not excluded per se. However, we excluded n = 19 persons who died during 2020 for whom health services were recorded at least a quarter after their death as these records were deemed implausible. We did not apply this selection criterion in the analyses with a longer follow-up as we only obtained data until the end of 2020 and persons who died during the observational period were already excluded. More information on how the death date was approximated with the help of the last recorded healthcare and long-term care services (we could only observe the quarter of death to ensure anonymity), can be found in the supplementary material of Henken et al. [6].

All costs used for balancing were aggregated during baseline in the respective healthcare sectors and calculated in 2020 Euros adjusted for inflation using the Gross Domestic Product price index [7]. Robust standard errors refer to “HC3” estimators as implemented in the sandwich R-package (version 3.1-0).

Supplementary table 1: Systemic diseases and associated ICD-10-GM codes

Disease	ICD-10-GM codes	ATC codes	Source for selection
Oral cancer	C00.−C14.-	-	Tranby et al. [8]
Cardiovascular disease	I61, I63, I64; I70; I21, I22, I24	-	Wake et al. [9]
Dementia	F00.-, F01.-, F02.-, F03.-, G30.-	N06DA, N06DX01	Bauer et al. [3]
Type 2 diabetes	E10.−E14.-[a]	A10A[a], A10B	Reitzle et al. [5]
COPD	J44.-	-	Buhl et al. [2]
Pneumonia	A06.5, A20.2, A22.1, A48.1, A70<br>B25.0, B37.1, B44.1, B45.0, B58.3, B59<br>J13.-,J14.-,J15.-, J16.0, J16.8, J17.1, J17.2, J17.3, J17.8, J18.0, J18.1, J18.2, J18.8, J18.9, J69.0, J69.1, J69.8, J85.1	-	Fassmer et al. [10]
Rheumatoid arthritis	M05.-, M06.-	H02A, H02B, L04AX03, M01CX01, L01BA01, L04AA13, M01CX02, P01BA01, P01BA02, L04AX01, L04AD01, L04AC03, L04AB, L04AA24, L01XC02, L04AC07	Callhoff et al. [4]

[a] Some of the diagnosis codes (E10; E13; E14) and ATC codes (A10A – insulin) are rather to be associated with type 1 diabetes or other diabetes types. These were used to differentiate between both diabetes types according to Reitzle et al. [5]. COPD = Chronic obstructive pulmonary disease; ATC = Anatomical Therapeutic Chemical.

Supplementary fig. 1 Study timeline and design

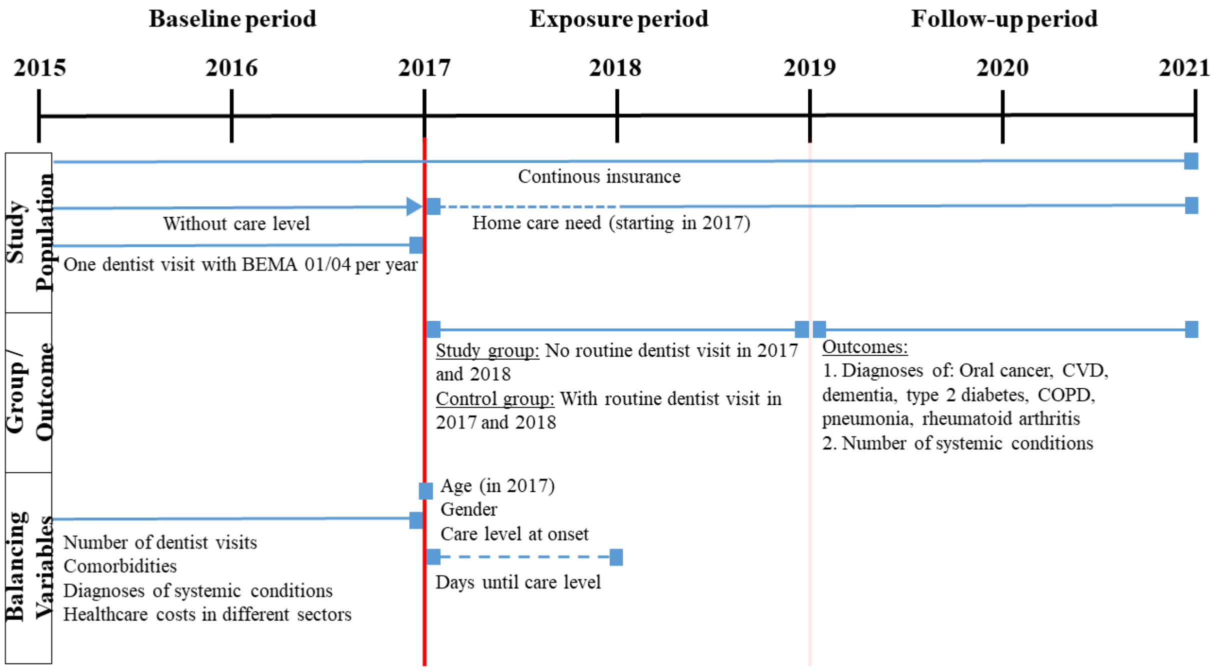

Notes. CVD: Cardiovascular disease; COPD: Chronic obstructive pulmonary disease

Supplementary fig 2: Flow-chart

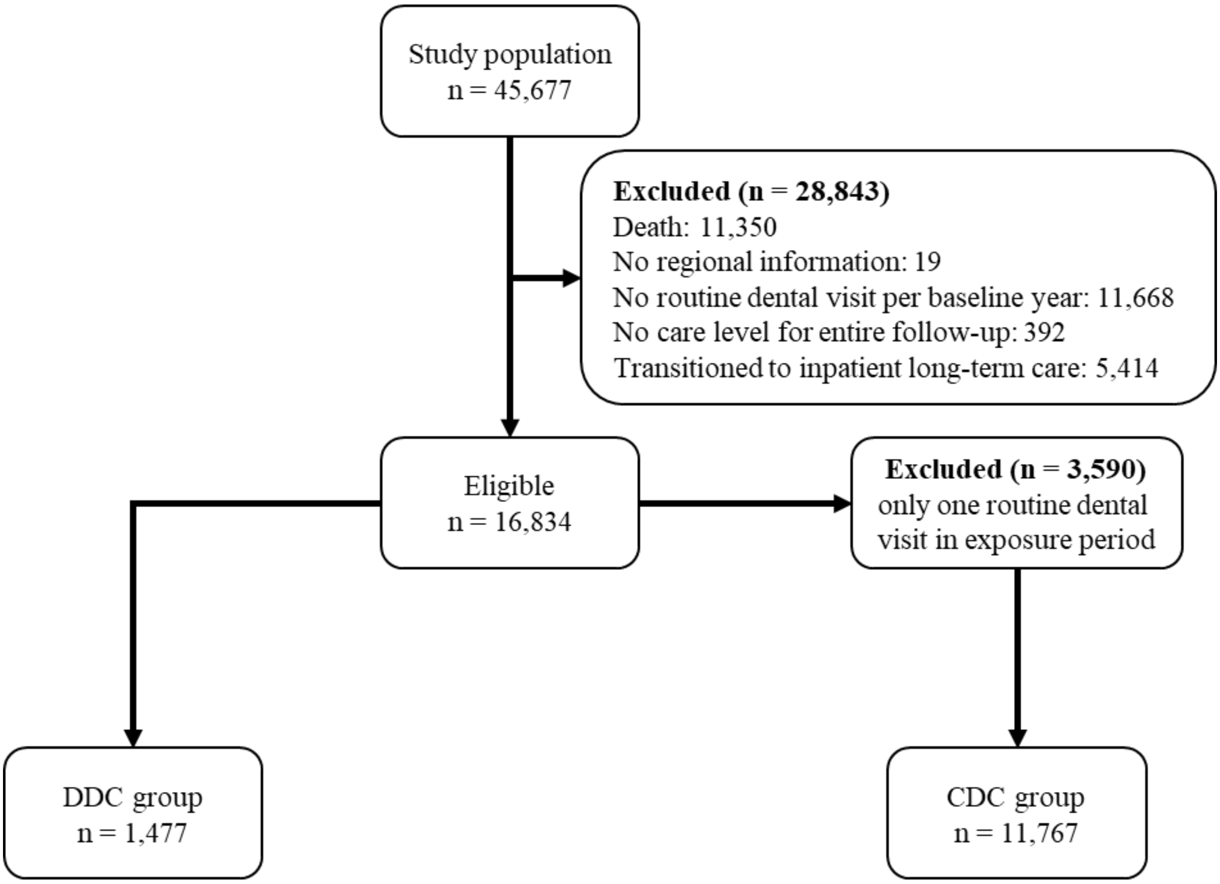

Notes. Study population: With long-term care need in 2017; DDC: Discontinued dental care (no routine dental service in 2017-2018); CDC: Continued dental care (routine dental service use in 2017 and 2018, respectively).

Supplementary fig. 3

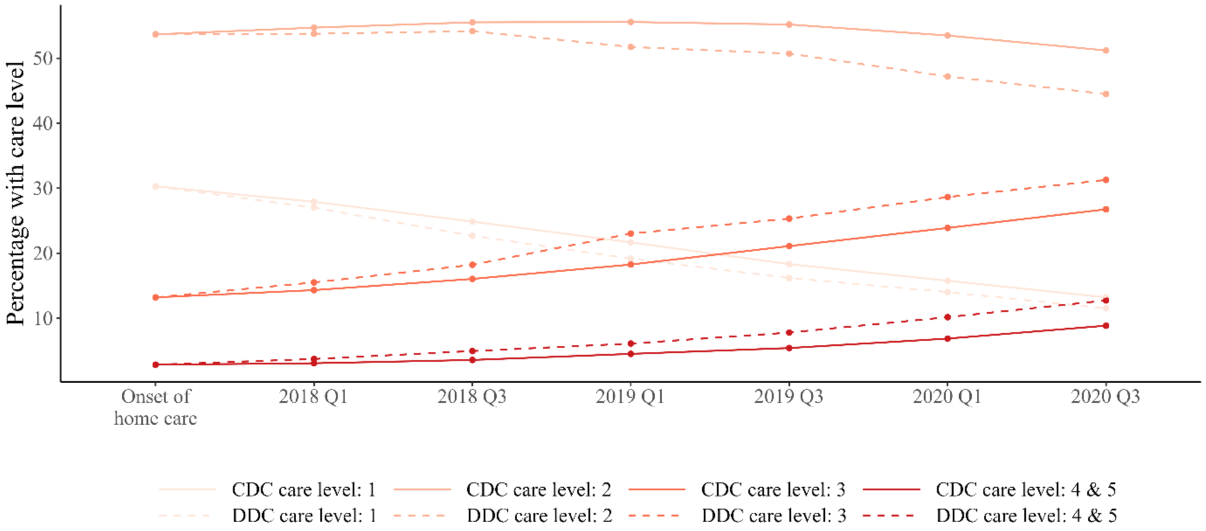

Notes. Progression of care levels in both groups. Values in CDC group were calculated using the entropy balancing weights.

References

1. Zimmermann T, Koenig A, Porzelt S, Schmage P, Konnopka C, Schellhammer S, et al. Interaction of Systemic Morbidity and Oral Health in Ambulatory Patients in Need of Home Care (InSEMaP): an observational study at the sector boundary between dental and general practice care in Germany. BMJ open. 2023;13(3):e063685. doi: 10.1136/bmjopen-2022-063685.
2. Buhl R, Wilke T, Picker N, Schmidt O, Hechtner M, Kondla A, et al. Real-World Treatment of Patients Newly Diagnosed with Chronic Obstructive Pulmonary Disease: A Retrospective German Claims Data Analysis. Int J Chronic Obstr Pulm Dis. 2022:2355-67. doi: 10.2147/COPD.S375190.
3. Bauer K, Schwarzkopf L, Graessel E, Holle R. A claims data-based comparison of comorbidity in individuals with and without dementia. BMC Geriatr. 2014;14:1-13. doi: 10.1186/1471-2318-14-10.
4. Callhoff J, Albrecht K, Marschall U, Strangfeld A, Hoffmann F. Identification of rheumatoid arthritis in German claims data using different algorithms: Validation by cross-sectional patient-reported survey data. Pharmacoepidemiol Drug Saf. 2023;32(5):517-25. doi: 10.1002/pds.5562.
5. Reitzle L, Ihle P, Heidemann C, Paprott R, Köster I, Schmidt C. Algorithmus zur Unterscheidung von Diabetes mellitus Typ 1 und Typ 2 bei der Analyse von Routinedaten. Gesundheitswesen. 2022;85(S02):199-26. doi: 10.1055/a-1791-0918.
6. Henken E, König H-H, Konnopka A, Behrens-Potratz A, Schellhammer S, Schmage P, et al. Utilization of dental services of older persons after onset of home care - an observational study from the InSEMaP research project based on German insurance claims data. BMC Geriatr. 2025;25:776. doi: 10.1186/s12877-025-06420-8.
7. Organisation for Economic Co-operation and Development. Economic References https://stats.oecd.org/Index.aspx?DataSetCode=HEALTH_ECOR (2024). Accessed February 5, 2024.
8. Tranby EP, Heaton LJ, Tomar SL, Kelly AL, Fager GL, Backley M, et al. Oral cancer prevalence, mortality, and costs in Medicaid and commercial insurance claims data. Cancer Epidemiol Biomarkers Prev. 2022;31(9):1849-57. doi: 10.1158/1055-9965.EPI-22-0114.
9. Wake M, Oh A, Onishi Y, Guelfucci F, Shimasaki Y, Teramoto T. Adherence and persistence to hyperlipidemia medications in patients with atherosclerotic cardiovascular disease and those with diabetes mellitus based on administrative claims data in Japan. Atherosclerosis. 2019;282:19-28. doi: 10.1016/j.atherosclerosis.2018.12.026.
10. Fassmer A, Spreckelsen O, Hoffmann F. Incidence of pneumonia in nursing home residents in Germany: results of a claims data analysis. Epidemiol Infect. 2018;146(9):1123-9. doi: 10.1017/S0950268818000997.
